# Novel Hybrid Peptide DEFB126 (1-39)-TP5 Inhibits LPS-Induced Inflammatory Responses and Oxidative Stress by Neutralizing LPS and Blocking the TLR4/MD2-NFκB Signaling Axis

**DOI:** 10.3390/antiox14091117

**Published:** 2025-09-14

**Authors:** Yuan Tang, Xuelian Zhao, Zetao Ding, Junyong Wang, Jing Zhang, Yichen Zhou, Marhaba Ahmat, Hao Wang, Yang Zhu, Baseer Ahmad, Zaheer Abbas, Dayong Si, Rijun Zhang, Xubiao Wei

**Affiliations:** 1 Laboratory of Feed Biotechnology, State Key Laboratory of Animal Nutrition and Feeding, College of Animal Science and Technology, China Agricultural University, Beijing 100193, China; ylyuan@cau.edu.cn (Y.T.); xlianzhao@cau.edu.cn (X.Z.); sy20243041014@cau.edu.cn (Z.D.); wangjy9722@cau.edu.cn (J.W.); zhangjing123@cau.edu.cn (J.Z.); yczhou51@alu.cau.edu.cn (Y.Z.); wh10@cau.edu.cn (H.W.); 20210204925@stu.qau.edu.cn (Y.Z.); zaheerabbas@cau.edu.cn (Z.A.); dayong@cau.edu.cn (D.S.); rjzhang@cau.edu.cn (R.Z.); 2 Institute of Microbiology, Xinjiang Academy of Agricultural Sciences, Urumqi 830091, China; malika511@xaas.ac.cn; 3 Xinjiang Laboratory of Special Environmental Microbiology, Urumqi 830091, China; 4 Faculty of Animal and Veterinary Sciences, Muhammad Nawaz Shareef University of Agriculture, Multan 25000, Pakistan; baseer.ahmad@mnsuam.edu.pk

**Keywords:** hybrid peptide, anti-inflammatory activity, antioxidant activity, molecule docking, TLR4/MD-2, ROS, lipopolysaccharide neutralization, NF-кB signaling pathway

## Abstract

Lipopolysaccharide (LPS), an essential structural molecule in the outer membrane of Gram-negative bacteria, is recognized as a principal trigger of inflammatory responses and oxidative stress. Thus, the control and clearance of LPS is essential to inhibit LPS-induced excessive inflammation, oxidative stress, and liver injury. In recent years, some native bioactive peptides, such as human β-defensin 126 (DEFB126) and thymopentin (TP5), have been reported to have inhibitory effects against LPS-induced inflammation and oxidative stress. However, the cytotoxicity, weak stability, and poor biological activity have hindered their practical application and clinical development. The development of novel hybrid peptides is a promising approach for overcoming these problems. In this study, we designed a novel hybrid peptide [DTP, DEFB126 (1-39)-TP5] that combines the active center of DEFB126 and full-length thymopentin (TP5). Compared to the parental peptides, DTP has a longer half-life, lower cytotoxicity, and greater anti-inflammatory and antioxidant activity. The anti-inflammatory and antioxidant effects of DTP were demonstrated in a murine LPS-induced sepsis model, which showed that DTP successfully inhibited the indicators associated with LPS-induced liver injury; decreased the contents of TNF-α, IL-6, and IL-1β; increased the level of glutathione (GSH); and improved the activities of catalase (CAT) and superoxide dismutase (SOD). Furthermore, our study revealed that the anti-inflammatory and antioxidant activities of DTP were associated with LPS neutralization, blockade of LPS binding to the Toll-like receptor 4/myeloid differentiation factor 2 (TLR4/MD-2) complex, reduction in reactive oxygen species content, and inhibition of the activation of the nuclear factor kappa-B (NF-кB) signaling pathway. These results elucidate the structural and functional properties of the peptide DTP, reveal its underlying molecular mechanisms, and shed light on its potential as a multifunctional agent for applications in agriculture, food technology, and clinical therapeutics.

## 1. Introduction

Lipopolysaccharide (LPS) is a major component of the cell wall of Gram-negative bacteria and is considered to be one of the main causes of inflammation [1,2]. In addition, LPS triggers the synthesis of reactive oxygen species (ROS) [3]; excessive production of ROS can lead to considerable stress [4,5]. Therefore, control and removal of LPS is essential to avoid LPS-induced hyperinflammation, oxidative stress, acute liver injury, and sepsis [6,7]. Antibiotics, such as polymyxin B, are considered the major therapeutic option to treat patients with LPS-induced inflammation and oxidative stress. Still, they are used as a last resort due to their neuro- and nephrotoxicity as well as drug resistance [8]. Therefore, there is an urgent need to design and develop new drugs that are more potent and safer.

In recent years, some bioactive peptides have been reported to inhibit LPS-induced inflammation and oxidative stress and thus are expected to be new anti-inflammatory and antioxidant agents [8,9,10,11,12,13,14]. Among them, human β-defensin 126 (DEFB126) and thymopentin (TP5) displayed enormous potential in the treatment of LPS-induced inflammation and oxidative stress [15,16,17].

β-defensins participate in antimicrobial defense and regulate both innate and adaptive immunity [18]. Human β-defensin 126 consists of 111 amino acids and contains three disulfide bonds [15]. The core functional region of human β-defensin 126 is the amino acid sequence 21–63 [15] (DEFB126 (21–63); abbreviated as DEFB126 peptide in this study). DEFB126 was found to exert anti-inflammatory activity by neutralizing LPS and manipulating the production of proinflammatory cytokines, including IL-1β, IL-6, and TNF-α [19,20]. Moreover, the β-defensin (DEFB) family has shown great efficacy in treating LPS-induced oxidative stress [21,22,23]. Therefore, DEFB126 can prevent or attenuate LPS-induced inflammation and oxidative stress.

TP5, the core active fragment of thymopoietin [24], exerts anti-inflammatory effects by suppressing the transcription factor nuclear factor kappa-B (NF-κB) and inhibiting mitogen-activated protein kinase (MAPK) signaling [16,25,26]. TP5 plays an important role in T-lymphocyte maturation and differentiation and regulates immune and the inflammatory responses [27,28,29]. Additionally, TP5 can promote the activity of superoxide dismutase (SOD), thus playing a significant role in inhibiting oxidative stress [27,30]. Overall, TP5 can be used for the treatment of inflammatory diseases and oxidative stress, due to its anti-inflammatory and antioxidant activities and low cytotoxicity.

Although DEFB126 and TP5 exhibit some anti-inflammatory and antioxidant potential, they face great challenges for practical application. The clinical development of DEFB126 has been hampered by its cytotoxicity [15]. TP5 exhibits minimal cytotoxicity, but its short half-life reduces its efficacy and bioavailability, thereby hindering its development [31]. The hybridization design strategy is an emerging protein engineering technology that combines functional fragments of different peptides to achieve integration of advantages [32,33,34,35]. It has been reported that DEFB126 (1-39) is the core active fragment of DEFB126 peptide, performing anti-inflammatory and antioxidant functions [15]. In this study, a hybrid peptide, DEFB126 (1-39)-TP5 (DTP), was designed by combining the DEFB126 (1-39) functional fragment with TP5. The hybrid peptide DTP was hypothesized to have greater anti-inflammatory and antioxidant activities, a longer half-life, and lower cytotoxicity than the parental peptides. Furthermore, we explored the anti-inflammatory and antioxidant activities of DTP in vivo and in vitro and elucidated its mechanism of action.

## 2. Materials and Methods

### 2.1. Hybrid Peptide Design

The hybrid peptide DTP was constructed by combining the active center of DEFB126 (1-39 amino acid truncates) with TP5. The primary sequence of the peptides was analyzed by the ExPASy Proteomics Server: https://www.expasy.org/resources/protparam (accessed on 1 August 2025). The three-dimensional (3D) structure of the hybrid peptide DTP was built using I-TASSER (https://zhanglab.ccmb.med.umich.edu/I-TASSER/ (accessed on 1 August 2025)).

### 2.2. Peptide Synthesis

The DEFB126, TP5, and DTP peptides were synthesized and purified (95% purity) by KangLong Biochemistry (Changzhou, China).

### 2.3. Circular Dichroism Analysis

The secondary structure of DTP was determined by circular dichroism (CD) spectroscopy. DTP was reconstituted in sterile water or 50% (*v*/*v*) trifluoroethanol (TFE) solution. CD spectra were acquired from 190 to 250 nm on a Jasco-810 spectropolarimeter (Jasco, Glasgow, UK).

### 2.4. Cell Culture

RAW 264.7 cells were maintained in DMEM medium containing 10% (*v*/*v*) fetal bovine serum and 1% (*v*/*v*) penicillin-streptomycin at 37 °C, 5% CO_2_.

### 2.5. Cell Viability Assay

The cytotoxicity of DTP, TP5, and DEFB126 was evaluated using the Cell Counting Kit-8 (CCK-8) Assay Kit (Dojindo Laboratories, Kumamoto, Japan) [1]. RAW264.7 cells (3 × 10^4^ cells/mL) were plated in 96-well plates in 100 μL DMEM overnight and then cultured in fresh medium containing a 2-fold concentration of peptide for another 24 h. Add 10 μL of CCK-8 solution to each well and incubate for 4 h away from light. Then, the optical density at 450 nm was measured using a microplate reader. Cell viability was determined by

Cell viability (%) = (OD_450 (sample)_/OD_450 (control)_) × 100% (1)

where OD_450 (sample)_ is the absorbance at 450 nm of the cells with peptides treated and OD_450 (control)_ is the absorbance at 450 nm of the cells without peptides treated.

### 2.6. Ex Vivo Stability of DTP

Half-life determination of DEFB126, TP5 and DTP (10 µg/mL) occurred in rat plasma. The above peptide solutions were incubated at 37 °C for varying lengths of time and then immediately transferred to pre-cooled test tubes containing 1 mL of acidic acetone (hydrochloric acid/acetone/H_2_O, *v*/*v*/*v* 1:40:5) to terminate the reaction. The concentration of the hydrochloric acid solution before dilution is 50 mM. The precipitate was subsequently separated by centrifugation at 20,000× *g* for 20 min at 4 °C. The obtained precipitates were dried in vacuo and then dissolved in 0.5 mL of 1 M acetic acid. Peptide analysis was conducted according to the protocol for the study of TP5 using HPLC [2]. Peptide half-lives were obtained by fitting a linear regression model based on logarithmic concentration-time.

### 2.7. Animal Model

Male C57BL/6 mice (6–8 weeks of age) were purchased from Charles River (Beijing, China). During the experiments, mice were housed in a specific-pathogen-free (SPF) environment with a temperature of 22 ± 1 °C and a relative humidity of 55 ± 10%. All the animal experiments were conducted with the approval of the Animal Care and Use Committee of China Agricultural University.

The mice were randomly divided into five groups of 12: control, LPS (*E. coli* O111: B4, Sigma-Aldrich, St. Louis, MO, USA), DEFB126 + LPS, TP5 + LPS, and DTP + LPS. The corresponding peptide (10 mg/kg) was injected intraperitoneally during the pretreatment stage. The control group was synchronized with the LPS group by injecting an equal amount of saline.

During the first 7 days of the experiment, each group received the above treatments once a day. On day 7, the LPS model group and the peptide pretreatment groups (DEFB126 + LPS, TP5 + LPS, and DTP + LPS) were injected intraperitoneally with 20 mg/kg of LPS to establish a sepsis model. The control group was injected with an equal volume of saline. Sixteen hours after the LPS injection, the mice were euthanized, and liver tissues and blood samples were collected. The serum was then separated by centrifugation and stored at −80 °C for measurement.

### 2.8. Histopathology and Immunohistochemistry

Mouse liver tissues were fixed with 4% paraformaldehyde, embedded in paraffin, and sliced continuously on a rotary slicer (Leica, RM2235, Nußloch, Germany) at a thickness of 5 μm. After dewaxing and rehydration, the tissues were subjected to Hematoxylin-Eosin (H&E) contrast staining. LPS-induced liver injury was evaluated according to a previous study. The liver injury score was graded on a 0- to 3-point scale: 0, no injury; 1, mild injury with cytoplasmic vacuolation; 2, moderate to severe injury consisting of extensive loss of intercellular borders and nuclear pyknosis; and 3, severe necrosis consisting of liver hemorrhage and neutrophil infiltration [4].

For immunohistochemical analysis, the sections were blocked with PBS containing 1% *w*/*v* bovine serum albumin for 1 h to seal the nonspecific binding sites, followed by incubation with anti-CD177^+^ antibody (1:100; Santa Cruz Biotechnology, Dallas, TX, USA) as a neutrophil marker, and finally the sections were fully rinsed with PBS. The sections were then incubated with horse-radish peroxidase (HRP)-conjugated rabbit anti-goat IgG (1:100; Jackson Immuno Research Laboratories INC, West Grove, PA, USA) for 1 h at 4 °C. After that, these sections were then sequentially stained with 3,3’-diaminobenzidine, stained with Harris hematoxylin, dehydrated with graded ethanol (70–100%), made transparent with xylene, and finally sealed with neutral gum.

### 2.9. Histopathology and Immunohistochemistry ELISA

The levels of tumor necrosis factor (TNF)-α, interleukin (IL)-6, and IL-1β in the serum of mice were detected using commercial ELISA kits (eBioscience, San Diego, CA, USA). Commercial reagent kits (Nanjing Jiancheng Bioengineering Institute, Nanjing, China) were used to detect the levels of serum alanine amino transferase (ALT) and aspartate amino transferase (AST).

### 2.10. The Detection of Reactive Oxygen Species (ROS) Generation

RAW264.7 cells (1 × 10^5^ cells/mL cell density) were incubated with LPS (100 ng/mL) at 37 °C for 1 h. After that, cells were treated with DTP at 37 °C for 12 h. The cells were then stained with 5 μM DCFH2-DA for 30 min at 37 °C in the dark. The cells were then washed extensively with PBS before analysis by fluorescence microscope and flow cytometry.

### 2.11. Neutralization of LPS

The LPS-neutralizing ability of the peptides was assessed through a quantitative Chromogenic End-point Tachypleus Amebocyte Lysate (CE TAL) assay (Xiamen Bioendo Technology Co., Xiamen, China). LPS (final concentration 1.0 U/mL; *E. coli*, O55:B5) was incubated with different concentrations of the peptides (final concentration 0 to 128 μg/mL) for 15 min at 37 °C. Then, the mixtures were incubated with TAL assay reagent at 37 °C for 6 min, and the optical density at 540 nm was measured.

### 2.12. Molecular Docking

The published crystal structure of mouse-derived Toll-like receptor 4/myeloid differentiation factor 2 (TLR4/MD-2) (PDB ID: 2Z64) was used as a receptor model, and the binding conformation and affinity of the DTP ligand to this complex were predicted and evaluated by molecular docking. The molecular docking modeling analysis was performed in a hydrophobic environment. ZDOCK 3.0.2 was used to produce the initial TLR4/MD-2-DTP complex. Using the rigid docking mode of ZDOCK 3.0, a total of 3600 initial docking conformations were generated for the TLR4/MD-2-DTP complex; the optimal conformations were screened based on the principle of lowest binding energy, and then flexible docking was performed through the RosettaDock 3.5 server, with 1000 independent sampling operations for each molecule.

### 2.13. Surface Plasmon Resonance (SPR)

SPR analysis was performed using a Biacore X100 Biomolecular Interactor (GE Healthcare, Pittsburgh, PA, USA). Running buffer (PBS containing 0.05% Tween 20) was flowed through the reaction cell (30 μL/min). DTP was immobilized on the surface of the CM5 sensing chip using the amino coupling method. To characterize the interaction of the peptide with the TLR4/MD-2 complex, peptide concentration gradients (0, 1.25, 2.5, 5, and 10 mM) were set up, and the sensing maps were acquired and subtracted from the reference signals of the blank channel (feed running buffer). After each experiment, the chip surface was regenerated with 10 mM Gly-HCl buffer (pH 2.5). Experimental data were processed by ProteOn Manager 2.0 software: the starting injection time point was aligned, baseline drift was corrected, and the sensing maps after reference deduction were fitted by a 1:1 binding model. The binding rate constant (Ka) and dissociation rate constant (Kd) for the peptide-TLR4/MD-2 interaction were calculated by fitting the binding and dissociation phase data in groups, and the equilibrium dissociation constant (K_D_) was calculated according to the following equation:

*K*_D_ = *K*_d_/*K*_a_ (2)

### 2.14. Western Blotting

Samples were fractionated into cytoplasmic and nuclear components using the NR-PER Nuclear and Cytoplasmic Protein Extraction Kit (Thermo Fisher Scientific Inc., Waltham, MA, USA). Protein levels in each fraction were determined via a bicinchoninic acid (BCA) assay (KeyGEN Biotech, Nanjing, China). Subsequently, protein fractions were separated by 10% SDS-polyacrylamide gel electrophoresis and transferred to methanol-activated PVDF membranes (Bio-Rad, Hercules, CA, USA). After membrane transfer, the membranes were blocked with 5% skimmed milk, followed by incubation with phosphorylated antibodies: p-IKK-β, p-IкB-α, p-NF-кB (p-p65), and GAPDH (Santa Cruz), with shaking overnight at 4 °C. After TBST washing, the membranes were incubated with HRP-labeled species-matched secondary antibody (HuaAn Biologicals, Hangzhou, China) for 60 min at room temperature. The density of specific proteins was quantified using the ChemiDoc MP imaging system (Bio-Rad, Hercules, CA, USA).

### 2.15. Statistics

All experimental data were based on the results of at least three independent replicated experiments and were expressed as mean ± standard deviation (mean ± SD). Statistical comparisons were carried out with *Student’s t* test using GraphPad Prism v6 software (La Jolla, CA, USA). Significance was claimed at *p* values ≤ 0.05; NS: *p* > 0.05, *: *p* ≤ 0.05, **: *p* ≤ 0.01, and ***: *p* ≤ 0.001.

## 3. Results

### 3.1. Peptide Design and Characterization

**Figure 1:**
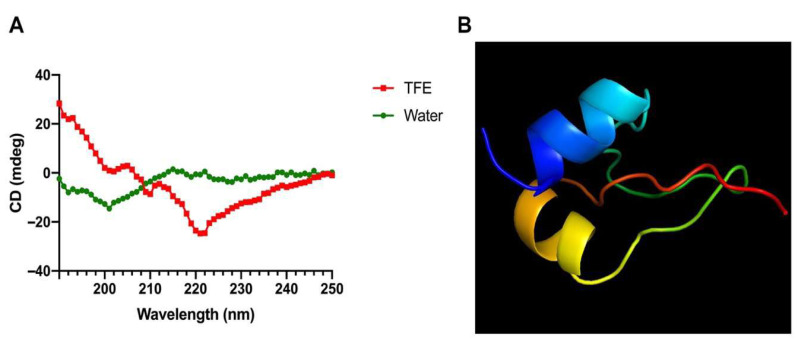
Overall structure of DTP. (**A**) CD spectra of DTP. The peptide samples were dissolved in sterile ultrapure water or a 50% TFE solvent system and measured by a Jasco J-810 spectrophotometer at 25 °C in the UV band of 190–250 nm. (**B**) 3D structure of DTP generated through I-TASSER.

Studies have shown that DEFB126 peptide is the core functional fragment of human β-defensin 126 and has excellent antibacterial and anti-inflammatory activities [15,20]. More specifically, DEFB126 (1-39) corresponds to the core functional fragment (21–59 amino acids) of human β-defensin 126, which contains three disulfide bonds associated with defensin function [15]. Therefore, we designed a novel hybrid peptide, DTP, by combining DEFB126 (1-39) with the TP5 peptide. However, to be honest, we are not sure whether the DEFB126 fragment will still form disulfide bonds after separation from the original protein. The sequences and key physicochemical parameters (molecular weight, net charge and mean hydrophobicity) of the newly designed peptide DTP and its parental peptides DEFB126 and TP5 are shown in Table 1. Linear (reduced) forms of DEFB126 and DTP peptides were used in the presented research. The DEFB126 peptide is a linear analog of the corresponding parent protein fragment. Circular dichroism (CD) determination of the secondary structure showed that DTP exhibited a typical α-helical conformation in 50% (*v*/*v*) TFE solution, while exhibiting an atactic convoluted conformation in aqueous environment (Figure 1A). I-TASSER prediction also showed that the main structure of DTP peptide is an α-helix structure (Figure 1B).

### 3.2. Cytotoxicity in RAW264.7 Macrophage Cells

**Figure 2:**
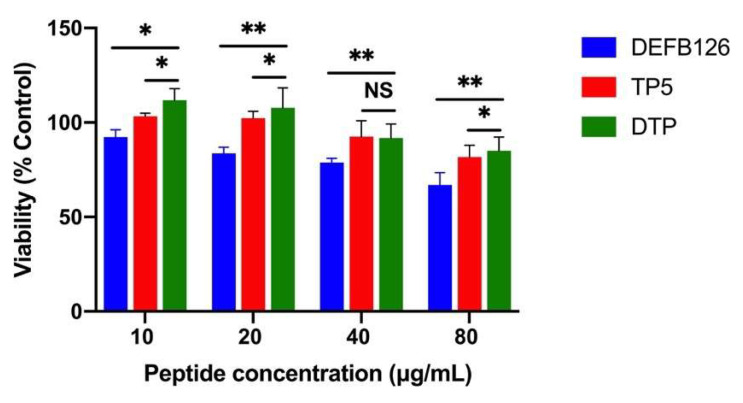
Cytotoxicity of DTP. RAW264.7 cells were treated with the peptides in a 2-fold series of concentrations for 24 h. Then, each well was incubated with 10 μL CCK-8 solution for 4 h away from light. The optical density at 450 nm was measured using a microplate reader. NS > 0.05, *: *p* ≤ 0.05, and **: *p* ≤ 0.01.

The cytotoxic activity of DTP and its parental peptides (DEFB126 and TP5) on RAW264.7 macrophages was evaluated by CCK-8 assay. The results showed that the cell survival rate was higher than 80% in all experimental groups after 24 h of treatment with 10 μg/mL peptide (Figure 2), indicating that this concentration was suitable for subsequent experiments. Notably, the cytotoxicity of DTP was significantly lower than DEFB126 and TP5.

### 3.3. Ex Vivo Stability of DTP in Plasma

**Figure 3:**
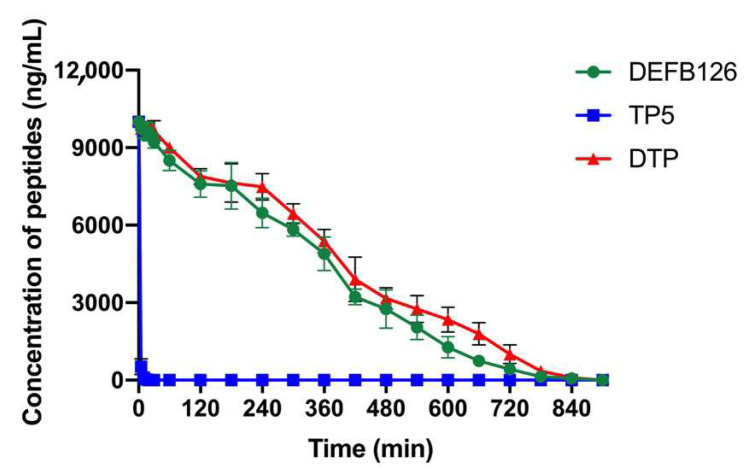
Plasma peptide concentrations (DEFB126, TP5, DTP) were quantified by HPLC.

The concentration of the peptides in plasma was determined by HPLC, and the results showed that the concentration of TP5 decreased to 5% after incubation in plasma for 5 min. Consistently, numerous studies have shown that TP5 is easily degraded by enzymes in plasma, so its stability in plasma is very poor [36,37,38]. In contrast to TP5, the engineered DTP peptide showed significantly improved stability, maintaining a half-life (t_1/2_) of 240 min during degradation studies. However, there was no statistical significance between DTP and DEFB126. (Figure 3 and Table 2).

### 3.4. Protective Effects of DTP Against LPS-Induced Liver Injury

**Figure 4:**
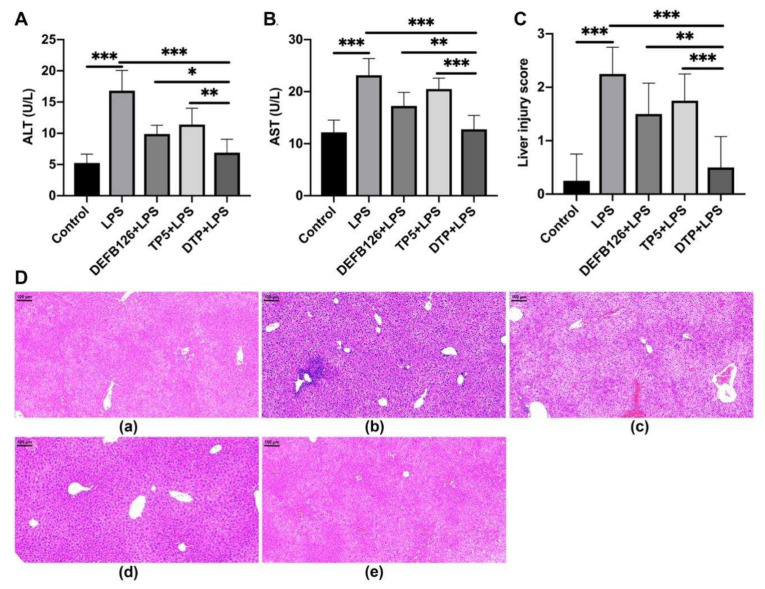
DTP exhibited protective effects on LPS-damaged liver tissue. Experimental mice were pretreated with 10 mg/kg peptides (DEFB126, TP5, DTP) or saline daily for 6 days. On day 6, LPS (10 mg/kg) was administered to treatment groups 1 h after final peptide/saline injection, with controls receiving saline alone. Sixteen hours after LPS treatment, mice were euthanized and assessed for differences in LPS-induced sepsis. ELISA was performed to detect alanine amino transferase (ALT) (**A**) and aspartate transaminase (AST) (**B**) in serum. (**C**) Liver injury scores. (**D**) Representative H&E-stained sections from the (**Da**) control, (**Db**) LPS, (**Dc**) DEFB126 + LPS, (**Dd**) TP5 + LPS, and (**De**) DTP + LPS groups. Bar, 100 μm. Data are given as the mean value ± SD from at least 3 biological replicates. *: *p* ≤ 0.05, **: *p* ≤ 0.01, and ***: *p* ≤ 0.001.

To characterize the protective effects of DTP against LPS-induced liver injury, the levels of ALT and AST, indicators of liver function, were determined by ELISA. As shown in Figure 4A,B, LPS resulted in elevated ALT and AST, whereas pretreatment with the peptides significantly attenuated these effects. In addition, the ALT and AST levels in the DTP-pretreated group were markedly lower than those in the TP5-pretreated group and DEFB126-pretreated group.

Liver histopathology assessment showed LPS-induced substantial tissue damage, including architectural distortion, extensive hemorrhage, hepatocellular necrosis, and marked inflammatory cell infiltration. However, DTP pretreatment reversed the severity of liver injury in LPS-treated mice (Figure 4D). These protective effects were confirmed by liver injury score analysis (Figure 4C), which showed that the DTP-pretreated group had a significantly lower liver injury score than the LPS-treated, TP5-pretreated, and DEFB126-pretreated groups.

### 3.5. The Effects of DTP on LPS-Induced Inflammatory Response

**Figure 5:**
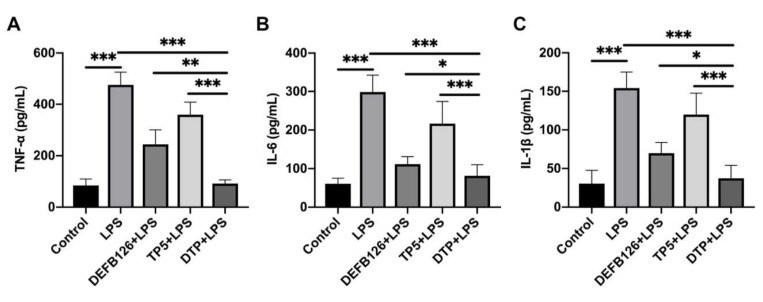
Anti-inflammatory effects of DTP against LPS-induced sepsis. ELISAs were performed to detect TNF-α (**A**), IL-6 (**B**), and IL-1β (**C**) in serum. Data are given as the mean value ± SD from at least 3 biological replicates. *: *p* ≤ 0.05, **: *p* ≤ 0.01, and ***: *p* ≤ 0.001.

To characterize the anti-inflammatory effect of DTP against LPS-induced sepsis, we quantified the concentrations of the inflammatory markers TNF-α (Figure 5A), IL-6 (Figure 5B), and IL-1β (Figure 5C) in mouse serum by ELISA. LPS caused a significant increase in serum TNF-α, IL-6, and IL-1β levels, whereas DTP significantly decreased the secretion of these proinflammatory cytokines. Moreover, serum TNF-α, IL-6, and IL-1β levels were significantly lower in the DTP-pretreated group than in the DEFB126-pretreated and the TP5-pretreated groups.

### 3.6. The Effects of DTP on LPS-Induced Oxidative Stress

**Figure 6:**
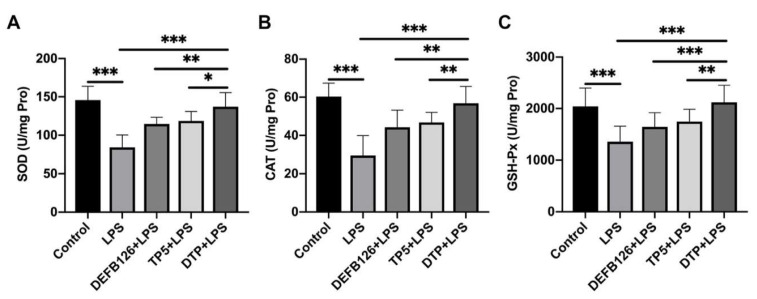
Effects of DTP on LPS-induced oxidative stress. The activities of SOD (**A**), CAT (**B**), and GSH-Px (**C**) in the liver were determined. Data are given as the mean value ± SD from at least 3 biological replicates. *: *p* ≤ 0.05, **: *p* ≤ 0.01, and ***: *p* ≤ 0.001.

To determine the antioxidant activity of DTP, the activities of SOD, CAT, and GSH-Px in the mouse liver were measured. As shown in Figure 6A–C, LPS led to significant decreases in liver SOD, CAT and GSH-Px activities, and DTP significantly reversed these effects. Furthermore, DTP was found to be more effective than DEFB126 or TP5 in maintaining liver SOD, CAT, and GSH-Px activities.

### 3.7. DTP Exerts Anti-Inflammatory and Antioxidant Activities by Neutralizing LPS, Binding to TLR4/MD-2, Reducing ROS Content and Inhibiting the Activation of the NF-κB Signaling Pathway

**Figure 7:**
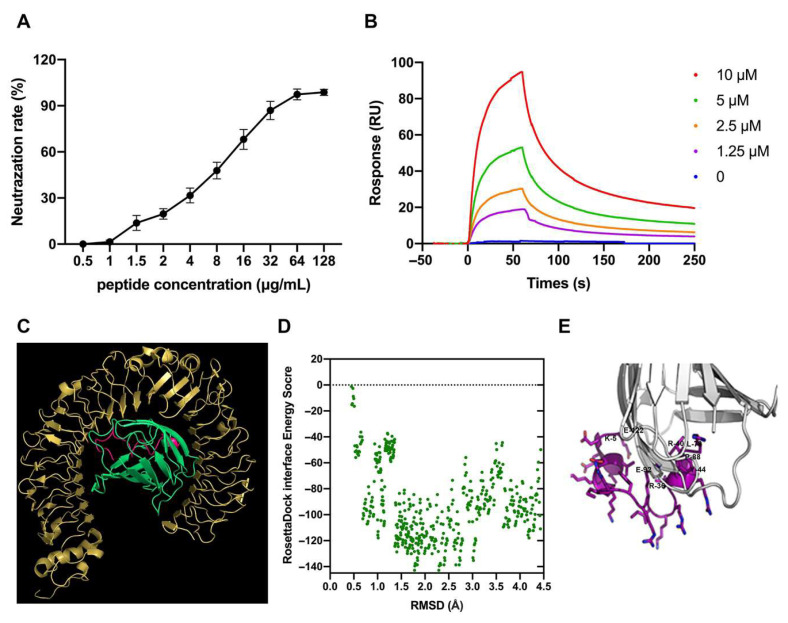
DTP neutralized LPS and blocked LPS binding to the TLR4/MD-2 complex. (**A**) The LPS neutralization activity of DTP was assessed by a chromogenic TAL assay. (**B**) TLR4/MD-2 was immobilized on a sensor chip, and the binding ability of DTP was analyzed by SPR. (**C**) Docking results of DTP on the TLR4/MD-2 complex. The crystal structure of TLR4 is displayed in yellow. The structure of MD-2 is colored green, and that of DTP is colored red. (**D**) Energy plot. Five hundred of 1000 decoy structures from the DTP-TLR4/md-2 docking study by RosettaDock in the liver were determined using diagnostic kits. (**E**) Close-up view of DTP binding to MD-2. The interacting residues between DTP and MD-2 are shown. The mean values ± standard deviation of at 3 independent experiments has been used to express the data.

To investigate the anti-inflammatory and antioxidant mechanisms of DTP, a CE TAL assay was performed. The results showed that DTP can directly neutralize LPS in a dose-dependent manner (Figure 7A). The 50% neutralization rate value of DTP was approximately 11.58 ± 2.07 μg/mL.

The binding of DTP to TLR4/MD-2 was examined via the SPR assay, which showed that DTP binding to the chip-bound protein exhibited a dose-dependent increase (Figure 7B). The calculated Ka and Kd values were 9.43 × 10^6^ s^−1^ and 2.17 × 10 M^−1^s^−1^, respectively, and the KD value was 2.30 × 10^−6^ M. To better understand how DTP interacts with the TLR4/MD-2 complex, the binding mode of DTP with the TLR4/MD-2 complex was analyzed by molecular docking. The interface energy score was used to reflect the binding affinity, and the results showed that the predicted binding energy of DTP was below 0, indicating that DTP could effectively bind to TLR4/MD-2 (Figure 7D). In addition, the predicted interface between TLR4/MD-2 and DTP (Figure 7C) was similar to that between LPS and TLR4/MD-2. DTP and LPS were found to bind to identical regions of the TLR4/MD-2 hydrophobic pocket, interacting with comparable residues (Figure 7E). This observation, consistent with SPR measurements, indicates that DTP achieves its therapeutic effects by competitively inhibiting LPS binding to the receptor complex.

**Figure 8:**
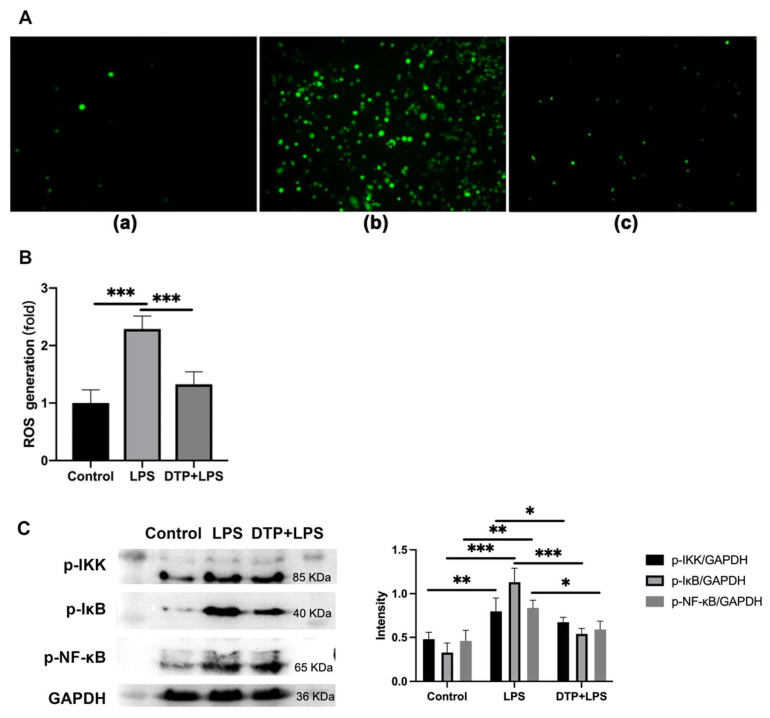
DTP suppressed the LPS-induced ROS generation and inhibited the NF-κB signaling pathway. RAW264.7 cells were co-incubated with LPS (100 ng/mL) for 1 h and then treated with or without peptides for 12 h. The cells were then stained with 5 μM DCFH2-DA for 30 min at 37 °C in the dark. ROS level was assessed with confocal microscopy (**A**) and flow cytometry (**B**). (**Aa**) control, (**Ab**) LPS, and (**Ac**) DTP + LPS groups. (**C**) Effect of DTP on the NF-кB signaling pathways. The phosphorylation levels of IKK-β, IκB-α, NF-κB, were detected by Western blot. Data are given as the mean value ± SD from at least 3 biological replicates. *: *p* ≤ 0.05, **: *p* ≤ 0.01, and ***: *p* ≤ 0.001.

ROS have been shown to perform a crucial role in regulating oxidative stress and inflammation. To explore whether DTP regulates oxidative stress and inflammatory responses through the ROS pathway, we examined macrophage ROS content by fluorescent staining and flow cytometry. The results showed that LPS significantly enhanced ROS generation, but DTP remarkably attenuated this promotion effect (Figure 8A,B). Analysis of NF-κB signaling pathway demonstrated that DTP counteracts LPS-induced effects. While LPS promoted phosphorylation of IKK-β, IκB-α and NF-κB subunits, DTP treatment significantly inhibited these phosphorylation processes (Figure 8C).

## 4. Discussion

Innate immune and oxidative regulatory responses play a crucial role as the first line of defense in recognizing and responding to a wide range of pathogens and small molecules [39,40,41,42]. As a potent inducer of inflammatory response dysregulation and redox imbalance, excessive LPS can result in acute liver damage [43,44] and sepsis [45]. Hence, controlling and removing LPS is essential to avoid excessive inflammation, oxidative stress, and organ damage. In recent years, bioactive peptides have shown great efficacy in modulating immune response and oxidative stress [46,47,48]. DEFB126 and TP5 have displayed some potential in the treatment of LPS-induced inflammation and oxidative stress [15,16]. Despite their therapeutic potential, DEFB126 and TP5 face significant limitations including cytotoxic effects, low metabolic stability, and insufficient anti-inflammatory/antioxidant properties. To overcome these challenges, hybridization has been proposed [49,50]. Based on this, we engineered a novel peptide by fusing full-length TP5 with the active region of DEFB126.

We aimed to design a peptide with enhanced anti-inflammatory and antioxidant activities, longer half-life than the parental peptide but less cytotoxic. We evaluated the cytotoxic activity of the hybrid peptide using the CCK-8 assay. DTP displayed superior safety profiles with lower cytotoxicity than its constituent peptides, potentially resulting from its rationally designed hydrophobic characteristics, in agreement with earlier reports [51]. In addition, we also detected the half-life of the hybrid peptide by HPLC. The results showed that the degradation profile and half-life were significantly prolonged.

LPS is a major cause of sepsis [52], which can cause excessive inflammation, oxidative stress, and liver injury [53]. Thus, the LPS-induced murine model of sepsis was used to evaluate the anti-inflammatory and antioxidant activities of DTP. LPS caused considerable liver injury, with increased ALT and AST, an altered hepatic architecture, extensive hemorrhage, necrosis of hepatocytes and infiltration of inflammatory cells. However, peptide pretreatment inhibited all these indices of LPS-induced liver injury (AST, ALT, histopathological changes and infiltration of activated neutrophils), and DTP showed greater potency than its parental peptides.

Cytokines are the key players in cell–cell communication in the immune system [54,55]. Cytokines, such as TNF-α, IL-6, and IL-1β, participated in amplifying the inflammatory cascade by triggering leukocyte aggregation and activation [56]. DTP’s capacity to counteract LPS-triggered inflammation was examined by measuring serum TNF-α, IL-6, and IL-1β levels. DTP treatment successfully inhibited LPS-mediated cytokine elevation and showed greater anti-inflammatory effects than its parental peptides.

LPS can cause considerable oxidative stress by promoting ROS production [57] and reducing endogenous antioxidant defenses [58]. GSH, as an important non-enzymatic antioxidant, can protect cells from oxidative damage by scavenging oxygen species [59,60,61]. In addition, SOD and CAT are important antioxidant enzymes that scavenge oxygen radical species and promote the decomposition of hydrogen peroxide in the liver [61]. In agreement with previous studies [62,63], we found that LPS treatment caused significant liver oxidative injury by decreasing the amount of GSH and the activities of CAT and SOD. However, treatment with peptides restored these perturbations in the antioxidant system. In addition, DTP peptide showed better potency than the parental peptides.

Taken together, these findings indicate DTP’s superior pharmacological profile, including greater anti-inflammatory/antioxidant efficacy, extended plasma half-life, and diminished cytotoxic effects compared to its parental peptides. To identify the anti-inflammatory and antioxidant mechanisms, a comprehensive and detailed analysis was conducted.

As a property of cationic peptides, LPS-neutralizing activity plays an important role in the inhibiting effects of cationic peptides in the LPS-induced inflammatory response and oxidative stress [64,65]. We found that DTP inhibited the tachypleus amebocyte lysate’s ability of LPS depending on the dose. The 50% binding rate value of DTP was approximately 11.58 ± 2.07 μg/mL. This suggests that DTP can inhibit the LPS-induced inflammatory response and oxidative stress by neutralizing LPS and then preventing its translocation across the cell membrane or intracellular accumulation.

TLR4 is the primary innate pattern recognition receptor for detecting and responding to LPS through accessory molecules, such as MD-2 [66]. Hence, preventing TLR4/MD-2 from binding to LPS is a potential mechanism for agents attenuating the LPS-induced inflammatory response and oxidative stress [67,68]. In this study, to investigate the TLR4/MD-2 complex binding ability of DTP, SPR binding assays were performed. The results confirmed that DTP could efficiently bind to the TLR4/MD-2 complex. Furthermore, molecular docking results indicated that the binding interface between TLR4/MD-2 and DTP was similar to that between LPS and TLR4/MD-2. Molecular docking analysis revealed that DTP and LPS occupy identical binding domains within the TLR4/MD-2 hydrophobic cavity, engaging comparable amino acid residues [69]. Collectively, these results suggest that DTP exerts its anti-inflammatory and antioxidant activities by blocking LPS from binding to the TLR4/MD-2 complex.

ROS has been proven to be an important cause of oxidative stress and inflammation [70,71]. In this study, the fluorescent staining and flow cytometry was performed to address whether DTP could regulate LPS-induced inflammation and oxidative stress through the ROS pathway. The results showed that LPS significantly enhanced ROS generation, while DTP remarkably attenuated this promotion effect. This finding emphasizes that DTP-mediated suppression of oxidative stress and inflammation is associated with reduced LPS-induced ROS accumulation.

The NF-κB signaling pathway plays a crucial role in host defenses through the regulation of inflammatory gene expression [72,73]. To elucidate DTP’s anti-inflammatory and antioxidant mechanisms, we examined its effects on NF-κB signaling. LPS treatment markedly enhanced IKK, IκB and NF-κB subunit phosphorylation, whereas DTP administration effectively attenuated these phosphorylation events.

## 5. Conclusions

**Figure 9:**
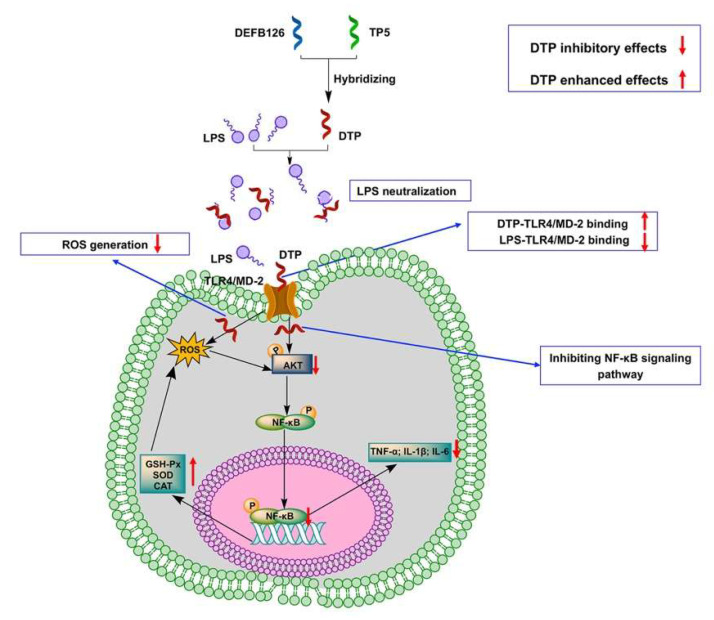
Schematic illustration of design of the novel hybrid peptide DTP to treat inflammation and oxidative stress induced by LPS.

In conclusion, we successfully designed a novel hybrid peptide DTP with both anti-inflammatory and antioxidant functions. Compared to the parental peptides, DTP has a longer half-life, lower cytotoxicity and greater anti-inflammatory and antioxidant activity. Our study revealed that the anti-inflammatory and antioxidant activities of DTP were associated with LPS neutralization, blockade of LPS binding to the Toll-like receptor 4/myeloid differentiation factor 2 (TLR4/MD-2) complex, reduction in reactive oxygen species content, and inhibition of the activation of the NF-кB signaling pathway (Figure 9). These findings offer substantial scientific foundation and methodological references for DTP’s potential utilization as a multifunctional candidate in diverse fields including agriculture, food science, and clinical applications. Moreover, DTP’s successful engineering establishes a viable strategy for developing innovative bioactive peptides with tailored therapeutic effects.

## Figures and Tables

**Table 1:** Key physicochemical parameters of parental and hybrid peptides.

Peptides	Sequence	Molecular Weight	Net Charge ^a^	H ^b^
DEFB126	NWYVKKCLNDVGICKKKCKPEEMHVKNGWAMCGKQRDCCVPAD	4957.9	+4	−0.638
TP5	RKDVY	679.8	+1	−1.680
DTP	NWYVKKCLNDVGICKKKCKPEEMHVKNGWAMCGKQRDCCRKDVY	5237.2	+6	−1.483

^a^ Net charge refers to the charge carried by peptides in a pH = 7 environment. ^b^ The mean hydrophobicity (H) is the total hydrophobicity (sum of all residue hydrophobicity indices) divided by the number of residues.

**Table 2:** Half-life of parental and hybrid peptides in plasma.

Peptides	DEFB126	TP5	DTP
t_1/2_ (min)	324.39 ± 53.09 ^a^	1.57 ± 0.35 ^b^	349 ± 69.17 ^a^

Data are given as the mean value ± SD from 3 biological replicates. ^a,b^ Means with different superscripts within the same row differ significantly (*p* ≤ 0.01).

## Data Availability

Data will be made available on request.
